# Supplementary material for: The Human Omnibus of Targetable Pockets
Source: J Cheminform. 2025 Dec 24;17:180. doi: 10.1186/s13321-025-01125-x (PMC12729103; doi:10.1186/s13321-025-01125-x)
Supplement: Supplementary file 2 — Supplementary Material 2. [file 13321_2025_1125_MOESM2_ESM.docx]

## **Supplement**

**Text S1.** List of excluded BioLiP ligand identifiers for “druglike ligand” filter to include only organic small molecules.

dna, rna, peptide, ACT, CA, CO, CO3, CU, FE, K, MG, MN, PO4, SO3, SO4, ZN

**Text S2.** Naive pocket filtering criteria.

AutoSite

Built-in score: None provided besides ranked pocket order.

Filter: Include only first five pockets.

CASTp

Built-in score: None provided besides ranked pocket order.

Filter: Include only first five pockets.

CavitySpace

Built-in score: Categorical pocket druggability classes.

Filter: Include only pockets labeled with “Strong” druggability.

Fpocket

Built-in score: Pocket druggability score ranging from 0 to 1.

Filter: Include only pockets with druggability score of at least 0.5.

LIGSITEcs

Built-in score: None provided besides ranked pocket order.

Filter: Include only first five pockets.

P2Rank

Built-in score: Pocket probability scores ranging from 0 to 1.

Filter: Include only pockets with probability score of at least 0.5.

PocketMiner

Built-in score: Per-residue pocket likelihood score ranging from 0 to 1; aggregated score cutoff of 0.7 already accounted for in method for generating predicted pockets.

Filter: Include only pockets with aggregated pocket likelihood score of at least 0.8.

**Text S3.** Hyperparameters explored for NN and CNN models.

NN hyperparameters

Batchsize: {32, 64, 128}

Dropout: {0, 0.2, 0.4, 0.5}

Learning rate: {0.01, 0.001}

Number of dense layers: {1, 3, 5}

Optimizer: {Adam, AdamW}

Hidden dimension varies as a function of input size, total number of dense layers, and layer number. See code on GitHub for more information.

CNN hyperparameters

Batchsize: {32, 64, 128, 256}

Dropout: {0, 0.2, 0.4, 0.5}

Learning rate: {0.01, 0.001, 0.0001}

Number of convolutional layers: {1, 2, 3}

Number of dense layers: {1, 3, 5}

Optimizer: {Adam, AdamW}

Pooling size: {64, 128, 256}

Convolutional filter size varies as a function of input size and layer number. Dense hidden dimension varies as a function of input size, total number of dense layers, and layer number. See code on GitHub for more information.

**Text S4.** KRAS switch I/II cryptic pocket residues.

Numbering from UniProt canonical sequence (accession P01116):

K5 L6 V7 E37 D38 S39 D54 I55 L56 M67 Q70 Y71 T74 G75

**Table S1.** Non-comprehensive list of pocket-finding methods from literature review conducted in 2023. See attached table.

**Table S2.** Training, validation, and testing sets for each of the constituent pocket-finding methods. See each constituent pocket-finding method’s original reference for more details on each dataset. Note that geometry-based and energy-based algorithms were not explicitly trained on a particular set of structures.

| **Method** | **Training set** | **Validation set** | **Testing set** |
| --- | --- | --- | --- |
| AutoSite | N/A – built on AutoDock4 affinity maps | AutoDock4 validated with Ligand-Protein Database (n = 188 structures) and PDBBind HIV protease complexes (n = 87 structures) | Astex Diverse Set (n = 85 structures) |
| CASTp | N/A – built on alpha shape method | N/A – built on alpha shape method | Monomeric enzymes from Laskowski *et al* (n = 51 structures) |
| CAVITY | N/A – built on probe sphere method | PDBBind 2007 (n = 1300 structures) | Q-SiteFinder holo dataset (n = 134 structures) and Q-SiteFinder apo dataset (n = 35) |
| Fpocket | N/A – built on alpha shape method | Non-redundant version of An *et al* dataset (n = 307 structures) | PocketPicker dataset (n = 48 structures), Cheng *et al* dataset (n = 20 structures), and Astex Diverse Set (n = 85 structures) |
| LIGSITEcs | N/A – built on grid method | N/A – built on grid method | Non-redundant Protein Ligand Database (n = 210 structures) and dataset based on Nissink *et al.* (n = 48 structures) |
| P2RANK | CHEN11 dataset (n = 251 structures) | Dataset based on Nissink *et al.* (n = 48 structures), non-redundant Protein Ligand Database (n = 210 structures), dataset based on Zhang *et al.* (n = 198 structures), Astex Diverse Set (n = 85 structures) | Subset of COACH test set (n = 420 structures) and database based on Schmidtke *et al.* (n = ~4000 structures) |
| PocketMiner | Dataset based on Zimmerman *et al.*, Cruz *et al.*, and known cryptic pockets (n = 37 structures) | Dataset based on Zimmerman *et al.*, Cruz *et al.*, and known cryptic pockets (n = 20 structures) | Curated examples of cryptic pockets (n = 35 structures) |

**Table S3.** Breakdown of proteins with experimentally-determined protein structures (“PDB”) and proteins with computationally-predicted protein structures (“AF2”) for which pocket annotations and predictions were generated when each method is run over all available structures for the canonical human proteome.

| **Method** | **Total # proteins** | **# AF2 proteins** | **# PDB proteins** |
| --- | --- | --- | --- |
| BioLIP | 4,437 | 0 | 4,437 |
| AutoSite | 20,171 | 20,127 | 7,087 |
| CASTp | 5,080 | 0 | 5,080 |
| CAVITY | 14,999 | 13,195 | 4,874 |
| Fpocket | 20,359 | 20,348 | 7,540 |
| LIGSITEcs | 7,083 | 0 | 7,083 |
| P2RANK | 16,996 | 16,066 | 7,080 |
| PocketMiner | 20,015 | 19,820 | 6,145 |

**Table S4.** Logistic regression hyperparameter tuning. For each combination of feature set and logistic regression penalty, the C value, L1 ratio, training set AUC, and validation set AUC are shown for the model with the best validation AUC out of all hyperparameter combinations assessed. Models were trained until convergence or a maximum of 500 iterations. There was no variation in performance across different random seeds. Feature set A is the per-residue pocket predictions from each of the constituent methods; Feature set B is the per-residue ESM2 embeddings; Feature set C is both the per-residue pocket predictions and per-residue ESM2 embeddings concatenated together.

| **Feature set** | **Penalty** | **C** | **L1 ratio** | **Train AUC** | **Val AUC** |
| --- | --- | --- | --- | --- | --- |
| A | None | N/A | N/A | 0.7793 | 0.7630 |
| A | L1 | 0.1 | N/A | 0.7779 | 0.7823 |
| A | L2 | 0.01 | N/A | 0.7695 | 0.7650 |
| A | Elastic Net | 0.1 | 0.9 | 0.7802 | 0.7754 |
| B | None | N/A | N/A | 0.9894 | 0.8849 |
| B | L1 | 1000 | N/A | 0.9894 | 0.8380 |
| B | L2 | 1 | N/A | 0.9894 | 0.8468 |
| B | Elastic Net | 1000 | 0.7 | 0.9894 | 0.8399 |
| C | None | N/A | N/A | 0.9894 | 0.8502 |
| C | L1 | 1 | N/A | 0.9894 | 0.8363 |
| C | L2 | 10 | N/A | 0.9894 | 0.8502 |
| C | Elastic Net | 0.1 | 0.3 | 0.9843 | 0.8520 |

**Table S5.** Neural network (NN and CNN) hyperparameter tuning. For each combination of feature set and neural network type (feedforward NN or convolutional NN), the number of convolutional layers, number of feedforward layers, batchsize, dropout probability, learning rate, optimizer, pooling size, training set AUC, and validation set AUC are shown for the model with the best validation AUC out of all hyperparameter combinations assessed. The AUCs shown are averaged over three identical models trained with different random seeds and are from the epoch with the highest validation AUC achieved in 50 epochs. Feature set A is the per-residue pocket predictions from each of the constituent methods; Feature set B is the per-residue ESM2 embeddings; Feature set C is both the per-residue pocket predictions and per-residue ESM2 embeddings concatenated together.

| **NN type** | **Feature set** | **# conv layers** | **# dense layers** | **Batchsize** | **Dropout** | **Learning rate** | **Optimizer** | **Pool size** | **Best mean train AUC** *(std.)* | **Best mean val AUC** *(std.)* |
| --- | --- | --- | --- | --- | --- | --- | --- | --- | --- | --- |
| NN | A | N/A | 3 | 32 | 0.5 | 0.001 | Adam | N/A | 0.8582 *(0.0107)* | 0.8745 *(0.0005)* |
| NN | B | N/A | 1 | 32 | 0.5 | 0.001 | AdamW | N/A | 0.9963 *(0.0048)* | 0.9208 *(0.0018)* |
| NN | C | N/A | 1 | 64 | 0.4 | 0.01 | Adam | N/A | 0.9995 *(0.0002)* | 0.9344 *(0.0019)* |
| CNN | A | 2 | 3 | 128 | 0.2 | 0.001 | Adam | Any | 0.8803 *(0.0076)* | 0.8748 (*0.0010)* |
| CNN | B | 1 | 5 | 128 | 0.0 | 0.001 | Adam | 64 | 0.9966 *(0.0022)* | 0.8907 *(0.0145)* |
| CNN | C | 1 | 5 | 128 | 0.0 | 0.001 | Adam | 128 | 0.9964 *(0.0026)* | 0.9072 *(0.0028)* |

**Table S6.** DCCcriterion using a threshold of 8A for top $N_{L}$ and top $N_{L}$+2 pockets generated by each method, where $N_{L}$ is the number of biologically-relevant ligands in each structure. DCCcriterion is defined as the percentage of pockets that have a center of mass within the threshold distance (here, 8A) of a biologically-relevant ligand; its values range from 0 to 100, with higher values indicating better performance. Any structures present in the *hotpocketNN* training or validation sets were omitted (401 structures removed from Human BioLiP dataset, 1 structure removed from Astex Diverse Set, 0 structures removed from PoseBusters dataset).

|  | **Astex Diverse Set** | | **PoseBusters** | | **Human BioLiP** | |
| --- | --- | --- | --- | --- | --- | --- |
|  | Top N | Top N+2 | Top N | Top N+2 | Top N | Top N+2 |
| *hotpocketNN*, ESM embs | **66.4** | **63.0** | **73.9** | **70.6** | 52.2 | 45.5 |
| *hotpocketNN*, combined | 55.7 | 53.3 | 58.4 | 54.4 | 42.5 | 37.7 |
| *hotpocketNN*, ESM embs, P2Rank only | 53.6 | 43.4 | 60.4 | 50.8 | 57.4 | **47.5** |
| *hotpocketNN*, combined, P2Rank only | 52.6 | 42.5 | 57.6 | 49.2 | 55.3 | 46.1 |
| AutoSite | 44.1 | 36.6 | 45.0 | 36.3 | 42.4 | 35.0 |
| CASTp | 36.7 | 30.5 | - | - | 36.1 | 29.3 |
| CAVITY | 42.9 | 33.3 | - | - | 27.4 | 22.6 |
| Fpocket | 50.5 | 39.1 | 49.4 | 39.6 | 44.3 | 35.6 |
| LIGSITEcs | 59.2 | 48.0 | 51.8 | 42.3 | 39.7 | 32.9 |
| P2Rank | 55.8 | 43.4 | 57.1 | 46.7 | **57.5** | 45.7 |
| PocketMiner | 58.8 | 56.4 | 56.4 | 56.0 | 44.5 | 43.1 |

**Table S7.** DCCcriterion using a threshold of 10A for top $N_{L}$ and top $N_{L}$+2 pockets generated by each method, where $N_{L}$ is the number of biologically-relevant ligands in each structure. DCCcriterion is defined as the percentage of pockets that have a center of mass within the threshold distance (here, 10A) of a biologically-relevant ligand; its values range from 0 to 100, with higher values indicating better performance. Any structures present in the *hotpocketNN* training or validation sets were omitted (401 structures removed from Human BioLiP dataset, 1 structure removed from Astex Diverse Set, 0 structures removed from PoseBusters dataset).

|  | **Astex Diverse Set** | | **PoseBusters** | | **Human BioLiP** | |
| --- | --- | --- | --- | --- | --- | --- |
|  | Top N | Top N+2 | Top N | Top N+2 | Top N | Top N+2 |
| *hotpocketNN*, ESM embs | **75.0** | **71.9** | **78.4** | **75.5** | 58.4 | 51.5 |
| *hotpocketNN*, combined | 60.9 | 58.3 | 64.2 | 60.2 | 49.0 | 43.8 |
| *hotpocketNN*, ESM embs, P2Rank only | 62.0 | 53.4 | 70.8 | 61.0 | **64.0** | **55.0** |
| *hotpocketNN*, combined, P2Rank only | 61.7 | 51.6 | 67.8 | 59.4 | 61.9 | 53.4 |
| AutoSite | 50.7 | 44.4 | 53.9 | 46.0 | 47.6 | 40.9 |
| CASTp | 42.4 | 36.5 | - | - | 41.8 | 35.4 |
| CAVITY | 57.1 | 50.0 | - | - | 34.6 | 29.7 |
| Fpocket | 55.6 | 45.6 | 57.6 | 47.3 | 49.8 | 41.7 |
| LIGSITEcs | 63.1 | 53.6 | 59.2 | 49.5 | 44.9 | 38.6 |
| P2Rank | 59.1 | 48.9 | 63.6 | 54.6 | 61.5 | 50.4 |
| PocketMiner | 72.8 | 71.2 | 69.2 | 69.5 | 54.3 | 53.1 |

**Table S8.** Summary of proteome-wide pockets after passing through the *hotpocketNN* filter (using Feature Set C: ESM2 embeddings and per-residue constituent method predictions) and the low-confidence AlphaFold2 filter.

| **Method** | **% candidate pockets accepted** | **# pockets** | **# proteins** | **Mean pockets per structure** *(std.)* | **Median pockets per structure** | **Mean pocket size** *(std.)* |
| --- | --- | --- | --- | --- | --- | --- |
| AutoSite | 12.52% | 288,293 | 16,953 | 5.71 *(7.43)* | 3 | 18.59 *(13.96)* |
| CASTp | 12.04% | 268,053 | 4,319 | 12.86 *(16.38)* | 8 | 9.28 *(17.81)* |
| CAVITY | 6.18% | 5,499 | 3,732 | 1.36 *(0.78)* | 1 | 45.89 *(36.29)* |
| Fpocket | 18.55% | 537,892 | 18,778 | 8.93 *(15.13)* | 5 | 11.56 *(7.83)* |
| LIGSITEcs | 35.26% | 58,859 | 5,443 | 1.76 *(0.92)* | 2 | 15.66 *(4.72)* |
| P2Rank | 49.22% | 369,656 | 16,299 | 6.70 *(13.53)* | 3 | 12.59 *(7.95)* |
| PocketMiner | 27.10% | 1,947,380 | 18,088 | 40.98 *(71.15)* | 20 | 11.00 *(0.00)* |

**Table S9.** Recovery of switch I/II cryptic pocket on various KRAS structures when predicted by *hotpocketNN*.

| **PDB ID** | **Ligand in cryptic pocket?** | **GDP / GTP bound** | **Year deposited in PDB** | **# pocket residues identified *(%)*** |
| --- | --- | --- | --- | --- |
| 6gj8 | Yes | GDP | 2018 | 14 *(100.00%)* |
| 3gft | No | GTP | 2009 | 1 *(7.14%)* |
| 5uk9 | No | GDP | 2017 | 13 *(92.86%)* |
| 6bp1 | No | GDP | 2017 | 7 *(50.00%)* |
| 6quu | No | GDP | 2019 | 14 *(100.00%)* |
| 7lz5 | No | GDP | 2021 | 8 *(57.14%)* |

**Table S10.** Recovery of orthosteric and allosteric pockets on various mOR structures when predicted by *hotpocketNN*. “Allosteric (inside)” is the allosteric pocket shown in 9bjk; “allosteric (outside)” is the allosteric pocket shown in 8k9l.

| **PDB ID** | **Year deposited in PDB** | **Pocket of interest** | **Ligand in pocket of interest?** | **# pocket residues identified *(%)*** |
| --- | --- | --- | --- | --- |
| 8k9k | 2023 | Orthosteric | No | 12 *(80.00%)* |
| 8k9k | 2023 | Allosteric (inside) | No | 14 *(73.68%)* |
| 8k9k | 2023 | Allosteric (outside) | No | 1 *(6.67%)* |
| 8k9l | 2023 | Orthosteric | No | 10 *(66.67%)* |
| 8k9l | 2023 | Allosteric (inside) | No | 11 *(57.89%)* |
| 8k9l | 2023 | Allosteric (outside) | Yes | 1 *(6.67%)* |
| 8ef5 | 2022 | Orthosteric | Yes | 14 *(93.33%)* |
| 8ef5 | 2022 | Allosteric (inside) | No | 9 *(47.37%)* |
| 8ef5 | 2022 | Allosteric (outside) | No | 6 *(40.00%)* |


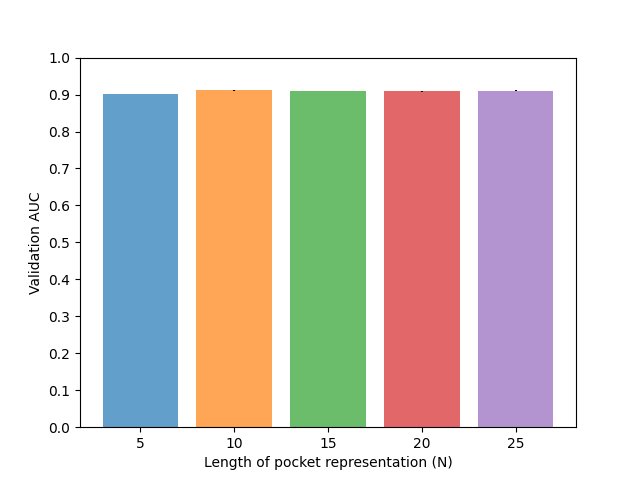


**Figure S1.** Ablation study varying $N$ (the size of the pocket representation input to *hotpocketNN*) for the final NN model architecture identified by hyperparameter tuning. All model hyperparameters are identical except for $N$. Performance shown is the mean maximum validation AUC achieved over 30 epochs over 10 random seeds. Standard error across the 10 runs for each model was calculated and is included as error bars.


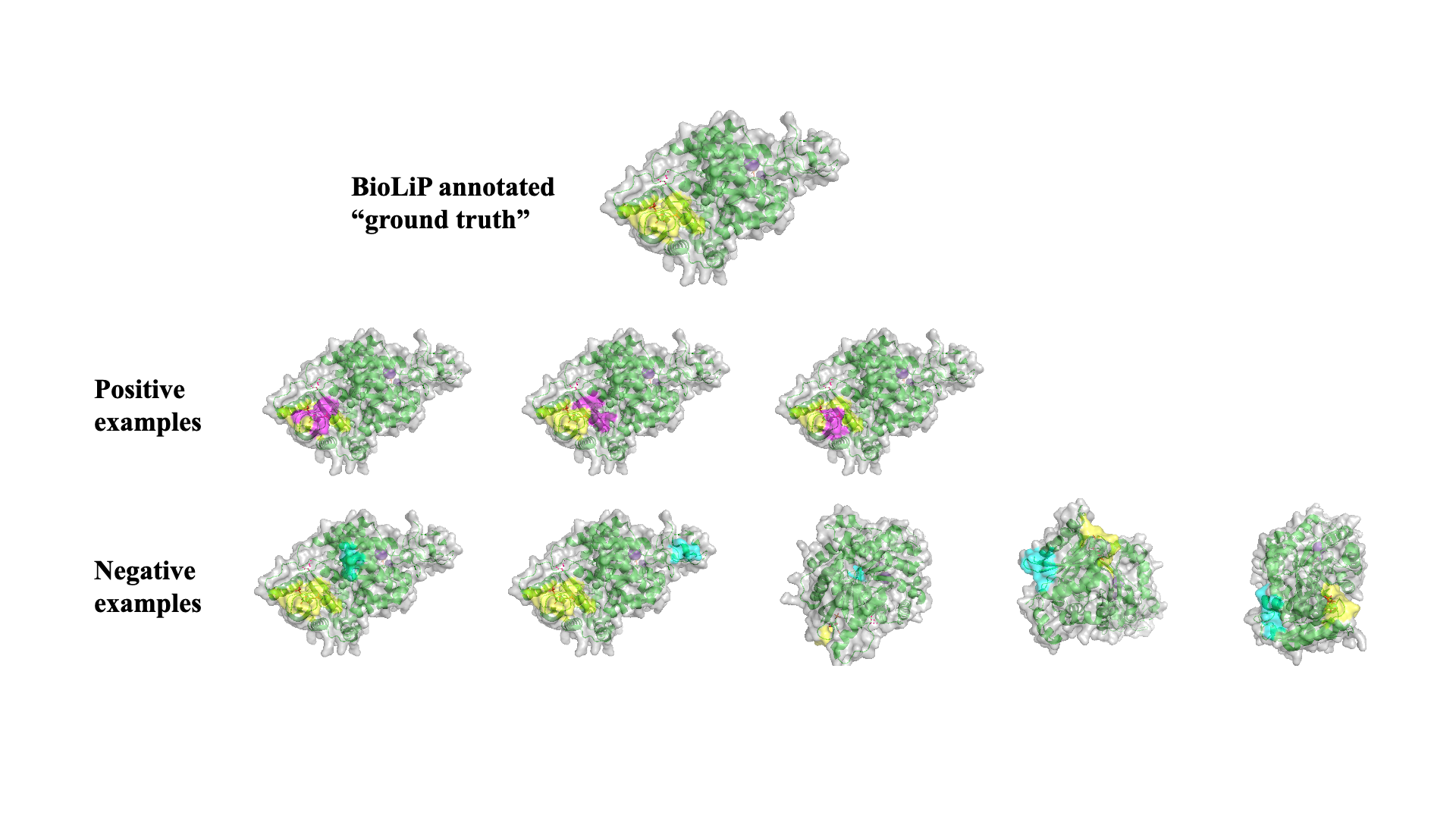


**Figure S2.** Schematic depicting process for generating positive and negative pocket examples from BioLiP known binding pocket annotations, to train, validate, and test the ML filtering method.


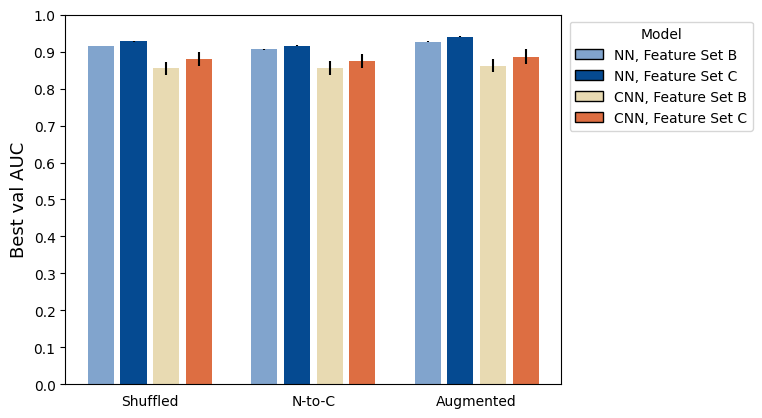


**Figure S3.** Performance of different pocket residue ordering schemes for both NNs and CNNs, and Feature Sets B and C. Average performance across different hyperparameter settings is shown with standard error.


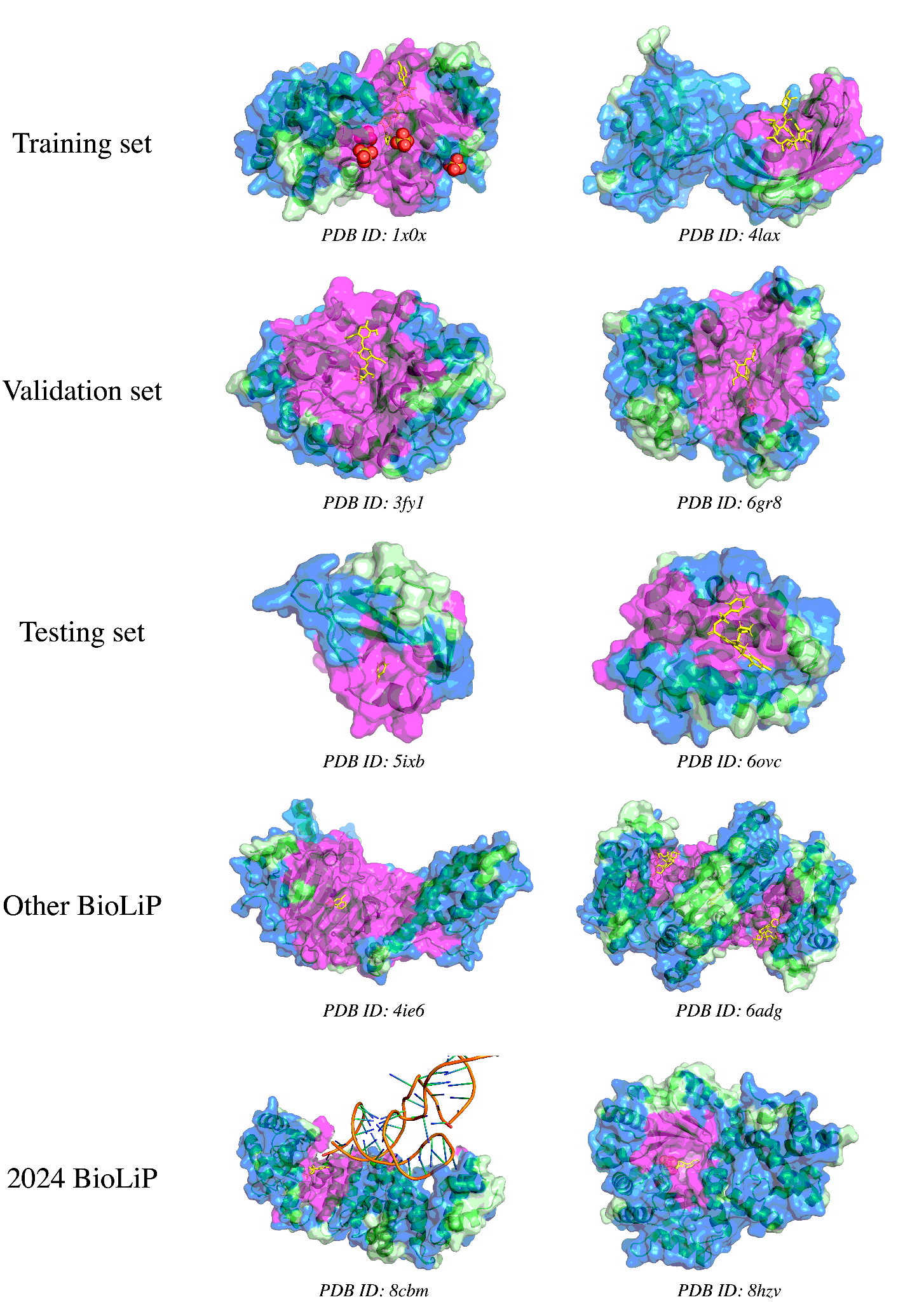


**Figure S4.** Visualizations of accepted and rejected candidate pockets on experimentally-determined structures from the PDB, using ESM2 embeddings as features for *hotpocketNN* ensembling and filtering method. From top row to bottom row, structures are taken from: the *hotpocketNN* training set, the *hotpocketNN* validation set, the *hotpocketNN* testing set, human protein structures with BioLiP annotations not included in the *hotpocketNN* train/val/test sets, and human protein structures with BioLiP annotations released in 2024 with low sequence identity to previously-seen structures. The surface of the protein structure is colored as follows: magenta for residues that are part of an accepted candidate pocket accepted by *hotpocketNN*, blue for residues that are part of a candidate pocket but not an accepted candidate pocket, and light green for residues that are not part of any candidate pockets. Biologically-relevant ligands are visualized in the structure as yellow sticks; non-biologically-relevant ligands are omitted. For each structure, we show only a single biological assembly; for the “2024 BioLiP” structures, we show only a single chain to better see the novel protein-ligand interaction.


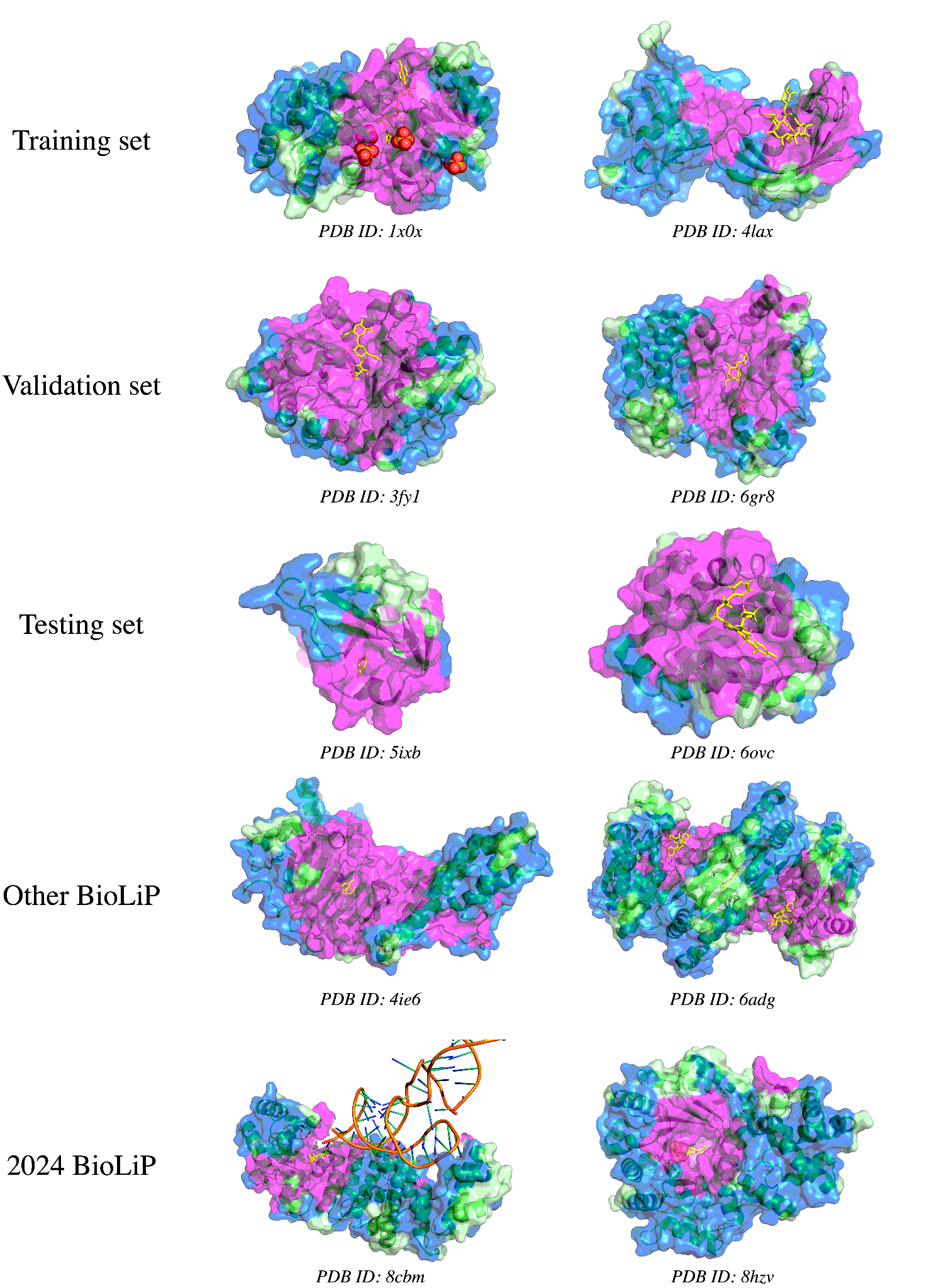


**Figure S5.** Visualizations of accepted and rejected candidate pockets on experimentally-determined structures from the PDB, using constituent method predictions and ESM2 embeddings as features for *hotpocketNN* ensembling and filtering method. From top row to bottom row, structures are taken from: the *hotpocketNN* training set, the *hotpocketNN* validation set, the *hotpocketNN* testing set, human protein structures with BioLiP annotations not included in the *hotpocketNN* train/val/test sets, and human protein structures with BioLiP annotations released in 2024 with low sequence identity to previously-seen structures. The surface of the protein structure is colored as follows: magenta for residues that are part of an accepted candidate pocket accepted by *hotpocketNN*, blue for residues that are part of a candidate pocket but not an accepted candidate pocket, and light green for residues that are not part of any candidate pockets. Biologically-relevant ligands are visualized in the structure as yellow sticks; non-biologically-relevant ligands are omitted. For each structure, we show only a single biological assembly; for the “2024 BioLiP” structures, we show only a single chain to better see the novel protein-ligand interaction.


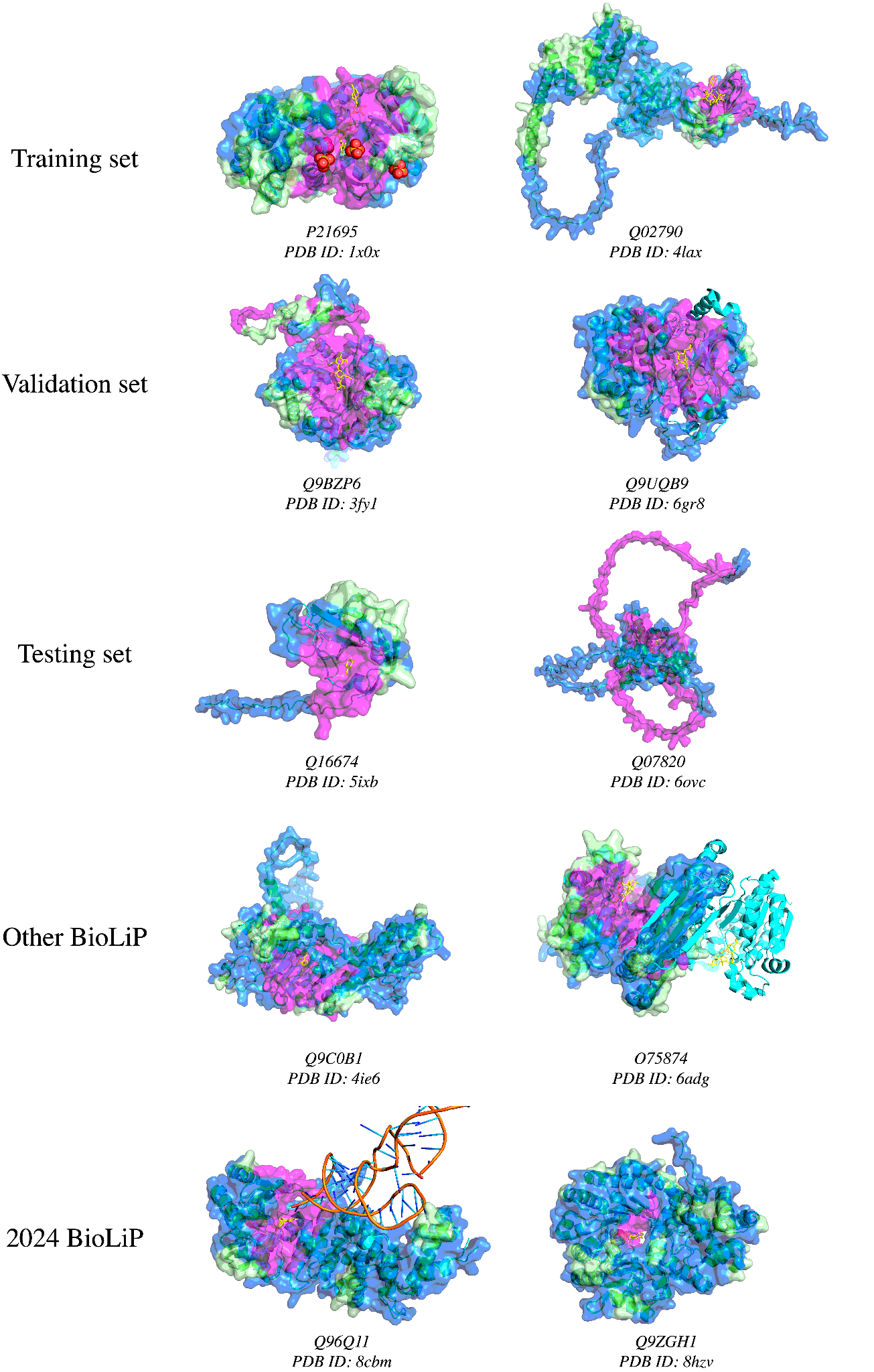


**Figure S6.** Visualizations of accepted and rejected candidate pockets on AlphaFold2-predicted protein structures, using ESM2 embeddings as features for *hotpocketNN* ensembling and filtering method. AlphaFold2-predicted structures (green ribbons) are shown aligned with their experimentally-determined PDB counterparts (cyan ribbons). Only the surface of the predicted structure is shown and pocket predictions were made on the predicted structure. The aligned ligands from the experimentally-determined structure are shown in yellow; these ligands are not a part of the AlphaFold2-predicted structures. The chains of the experimentally-determined structures are the same as in **Figure 5**. From top row to bottom row, structures are taken from: the *hotpocketNN* training set, the *hotpocketNN* validation set, the *hotpocketNN* testing set, human protein structures with BioLiP annotations not included in the *hotpocketNN* train/val/test sets, and human protein structures with BioLiP annotations released in 2024 with low sequence identity to previously-seen structures. The surface of the protein structure is colored as follows: magenta for residues that are part of an accepted candidate pocket accepted by *hotpocketNN*, blue for residues that are part of a candidate pocket but not an accepted candidate pocket, and light green for residues that are not part of any candidate pockets.


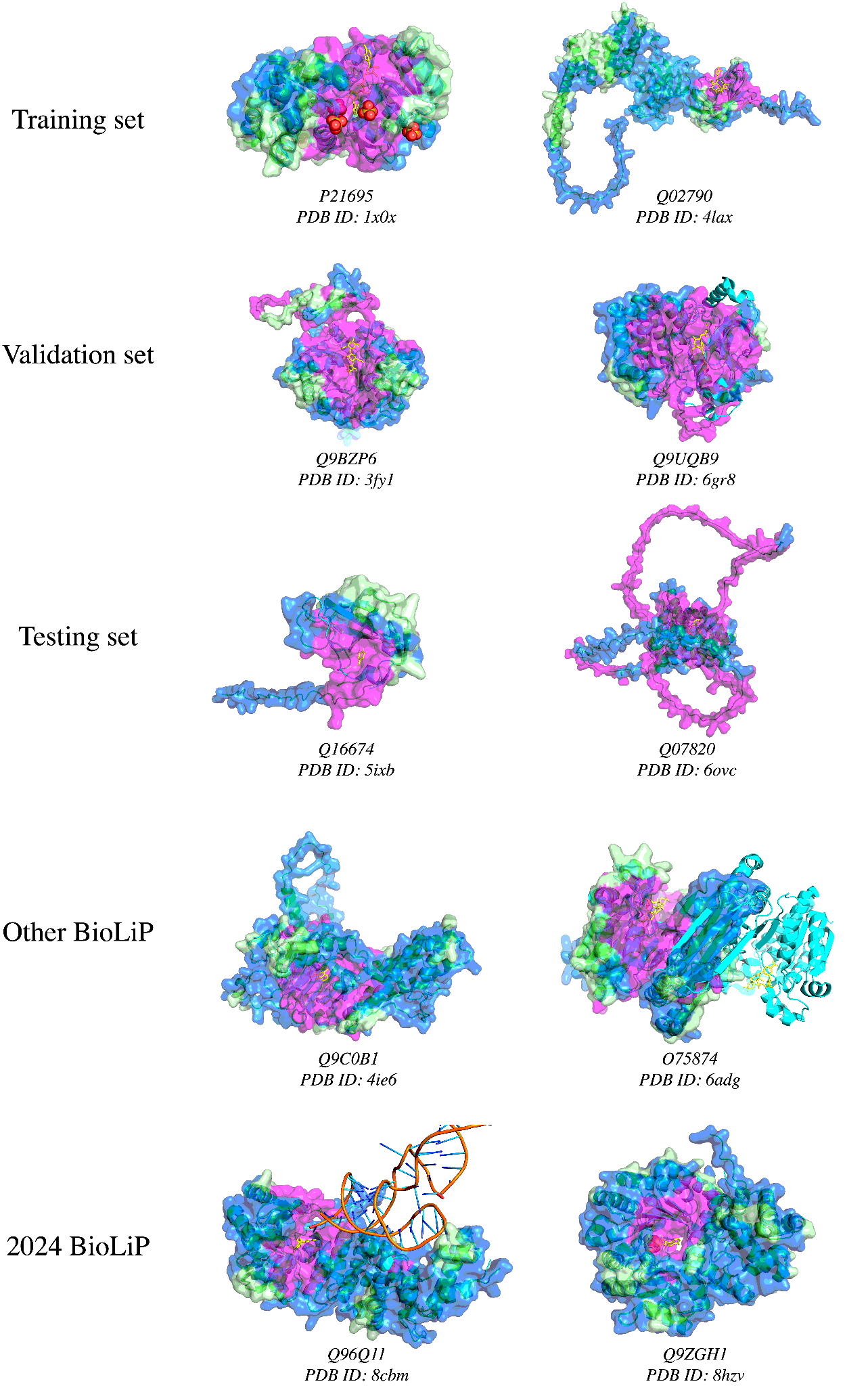


**Figure S7.** Visualizations of accepted and rejected candidate pockets on AlphaFold2-predicted protein structures, using constituent method predictions and ESM2 embeddings as features for *hotpocketNN* ensembling and filtering method. AlphaFold2-predicted structures (green ribbons) are shown aligned with their experimentally-determined PDB counterparts (cyan ribbons). Only the surface of the predicted structure is shown and pocket predictions were made on the predicted structure. The aligned ligands from the experimentally-determined structure are shown in yellow; these ligands are not a part of the AlphaFold2-predicted structures. The chains of the experimentally-determined structures are the same as in **Figure S3**. From top row to bottom row, structures are taken from: the *hotpocketNN* training set, the *hotpocketNN* validation set, the *hotpocketNN* testing set, human protein structures with BioLiP annotations not included in the *hotpocketNN* train/val/test sets, and human protein structures with BioLiP annotations released in 2024 with low sequence identity to previously-seen structures. The surface of the protein structure is colored as follows: magenta for residues that are part of an accepted candidate pocket accepted by *hotpocketNN*, blue for residues that are part of a candidate pocket but not an accepted candidate pocket, and light green for residues that are not part of any candidate pockets.


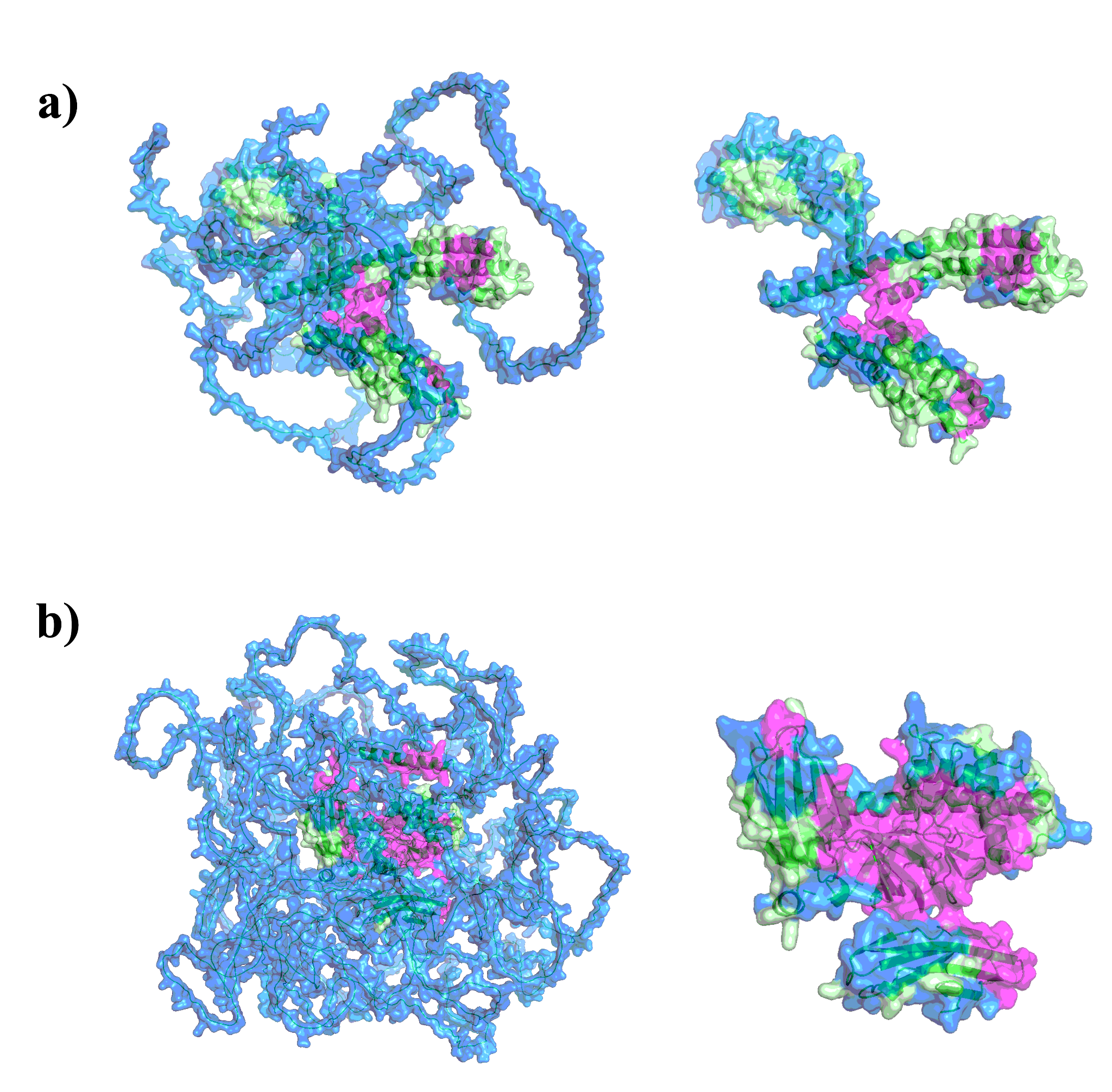


**Figure S8.** Visualizations of accepted and rejected candidate pockets on AlphaFold2-predicted protein structures with an average per-residue confidence that is **a)** “low” (average pLDDT between 50 and 70; UniProt ID: Q92953), or **b)** “very low” (average pLDDT less than 50; UniProt ID: Q86TB3), using ESM2 embeddings as features for *hotpocketNN* ensembling and filtering method. Neither protein has an experimentally-determined protein structure. The surface of the protein structure is colored as follows: magenta for residues that are part of an accepted candidate pocket accepted by *hotpocketNN*, blue for residues that are part of a candidate pocket but not an accepted candidate pocket, and light green for residues that are not part of any candidate pockets. Both the full predicted protein structure (left) and the subset of the structure that is not low-confidence (pLDDT greater than 70) (right) are shown.


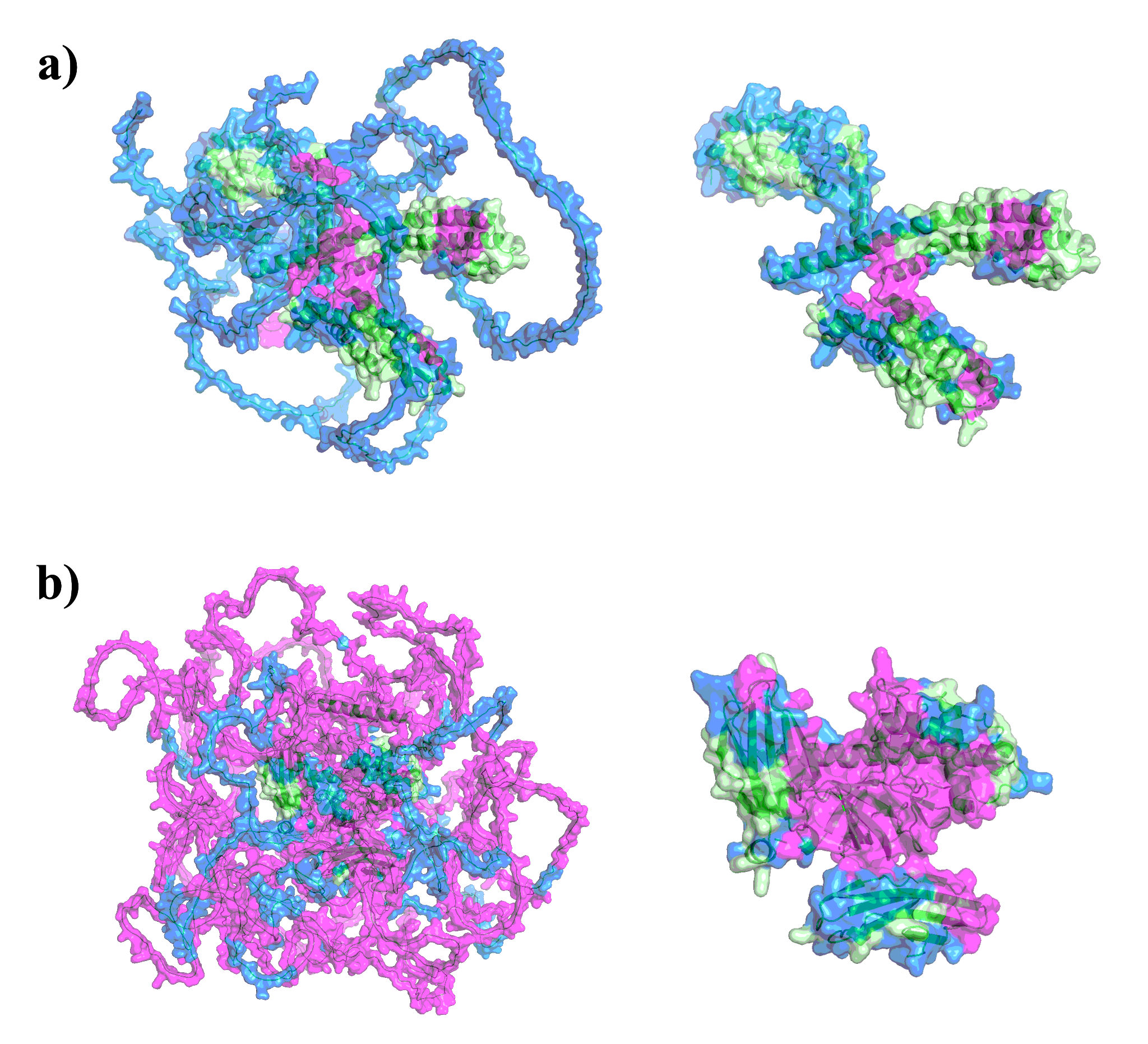


**Figure S9.** Visualizations of accepted and rejected candidate pockets on AlphaFold2-predicted protein structures with an average per-residue confidence that is **a)** “low” (average pLDDT between 50 and 70; UniProt ID: Q92953), or **b)** “very low” (average pLDDT less than 50; UniProt ID: Q86TB3), using constituent method predictions and ESM2 embeddings as features for *hotpocketNN* ensembling and filtering method. Neither protein has an experimentally-determined protein structure. The surface of the protein structure is colored as follows: magenta for residues that are part of an accepted candidate pocket accepted by *hotpocketNN*, blue for residues that are part of a candidate pocket but not an accepted candidate pocket, and light green for residues that are not part of any candidate pockets. Both the full predicted protein structure (left) and the subset of the structure that is not low-confidence (pLDDT greater than 70) (right) are shown.

**
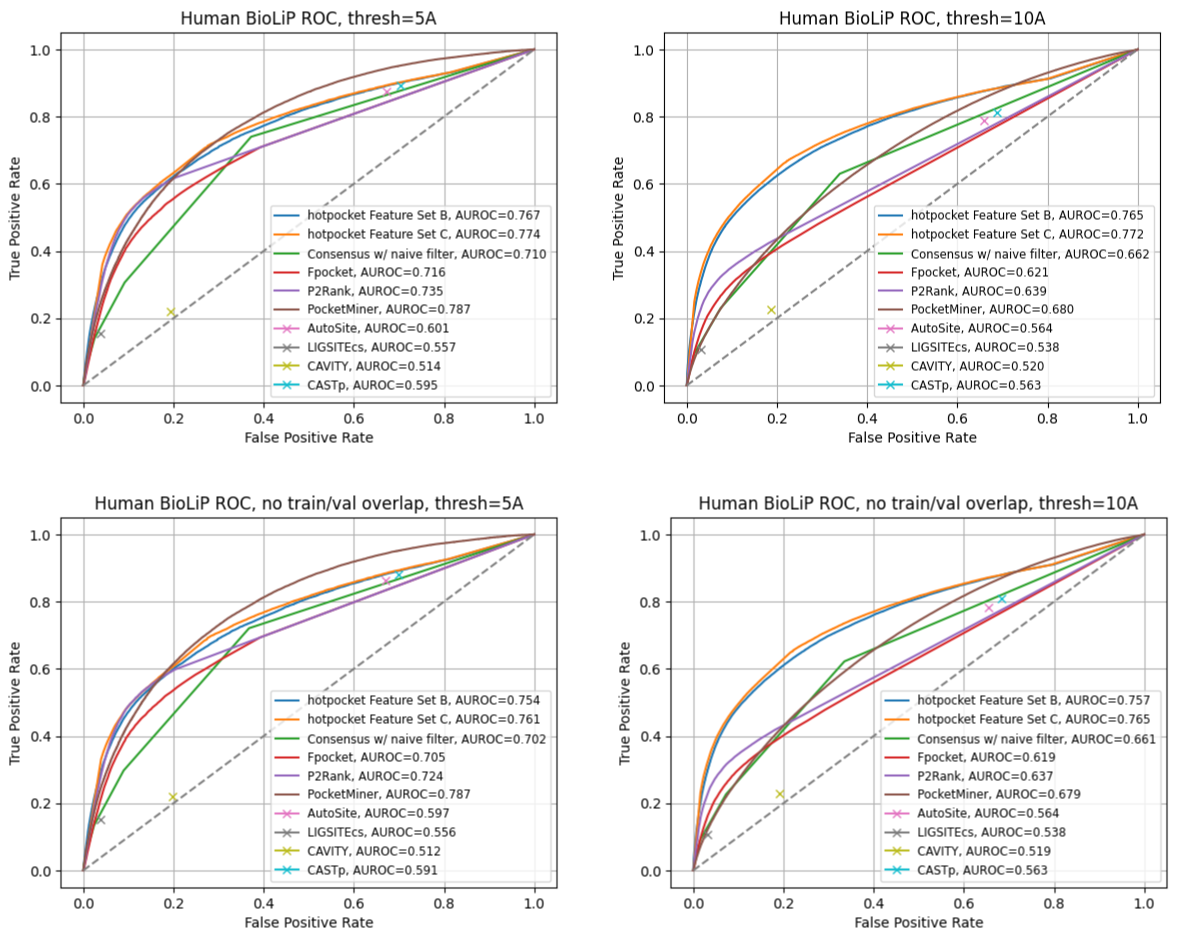
**

**Figure S10.** ROC curves and AUROCs for per-residue scoring performance of *hotpocketNN*, naive filter across union of constituent methods, and each constituent pocket-finding method individually across the Human BioLiP dataset. Each residue was labeled as a binding residue if it was within 5 angstroms (left) or 10 angstroms (right) of a relevant ligand. For the *hotpocketNN* models, Fpocket, P2Rank, and PocketMiner, each residue’s predicted score for being a member of a pocket was the maximum of all predicted pockets of which it was a member. AutoSite, LIGSITEcs, CAVITY, and CASTp do not provide pocket scores; for these methods, each residue’s predicted score for being a member of a pocket was 1 if it is part of any predicted pocket for the structure, or 0 otherwise. The top row shows results when all structures in all datasets are included, including structures present in the training and validation sets for the *hotpocketNN*. The bottom row shows results when structures present in the training and validation sets for the *hotpocketNN* are excluded. CAVITY and CASTp did not have predictions available for any PoseBusters structures. Feature Set B is the per-residue ESM2 embeddings; Feature Set C is both the per-residue pocket predictions and per-residue ESM2 embeddings concatenated together.


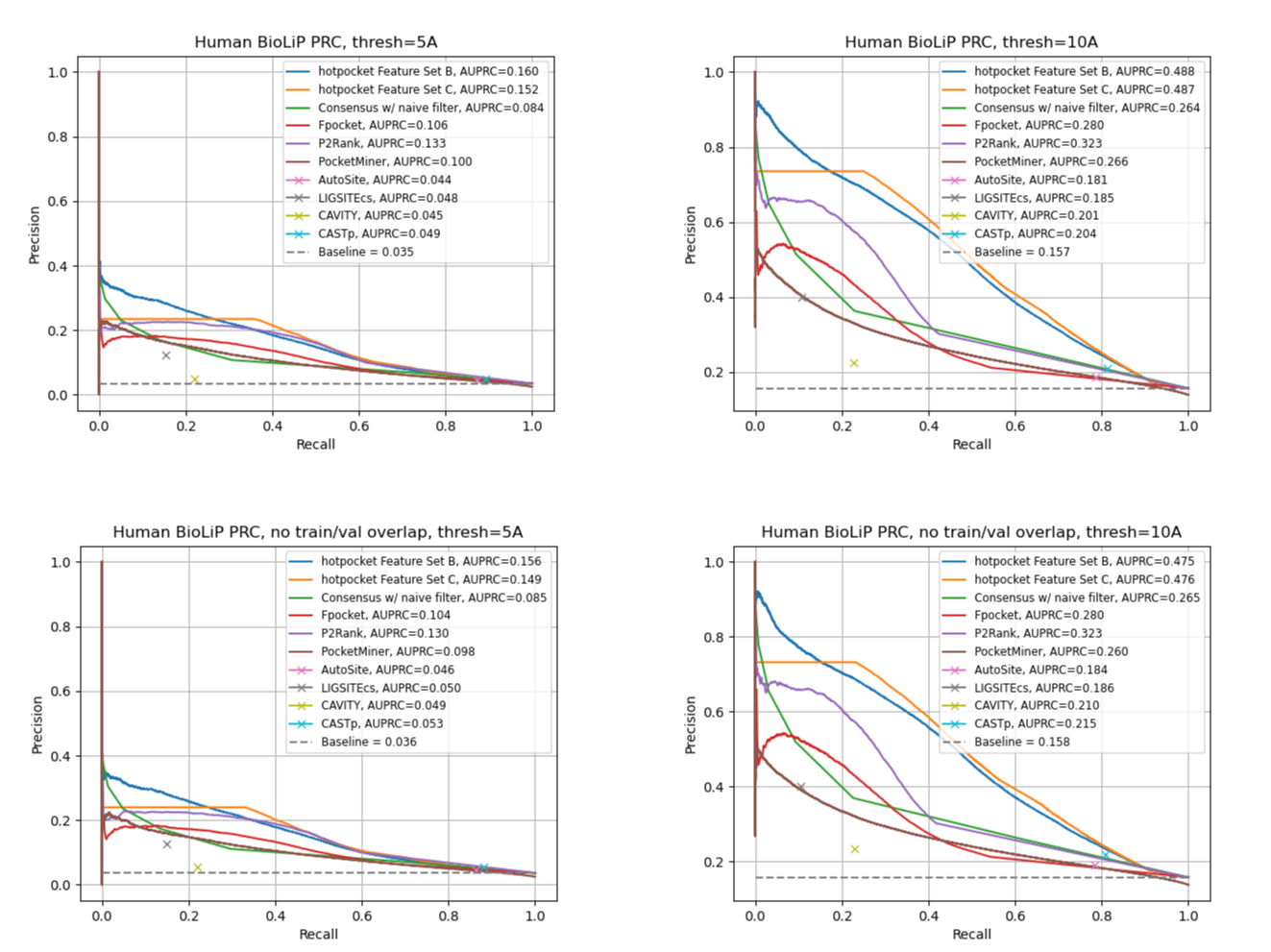


**Figure S11.** PRC curves and AUPRCs for per-residue scoring performance of *hotpocketNN*, naive filter across union of constituent methods, and each constituent pocket-finding method individually across the Human BioLiP dataset. Each residue was labeled as a binding residue if it was within 5 angstroms (left) or 10 angstroms (right) of a relevant ligand. For the *hotpocketNN* models, Fpocket, P2Rank, and PocketMiner, each residue’s predicted score for being a member of a pocket was the maximum of all predicted pockets of which it was a member. AutoSite, LIGSITEcs, CAVITY, and CASTp do not provide pocket scores; for these methods, each residue’s predicted score for being a member of a pocket was 1 if it is part of any predicted pocket for the structure, or 0 otherwise. The top row shows results when all structures in all datasets are included, including structures present in the training and validation sets for the *hotpocketNN*. The bottom row shows results when structures present in the training and validation sets for the *hotpocketNN* are excluded. CAVITY and CASTp did not have predictions available for any PoseBusters structures. Feature Set B is the per-residue ESM2 embeddings; Feature Set C is both the per-residue pocket predictions and per-residue ESM2 embeddings concatenated together.


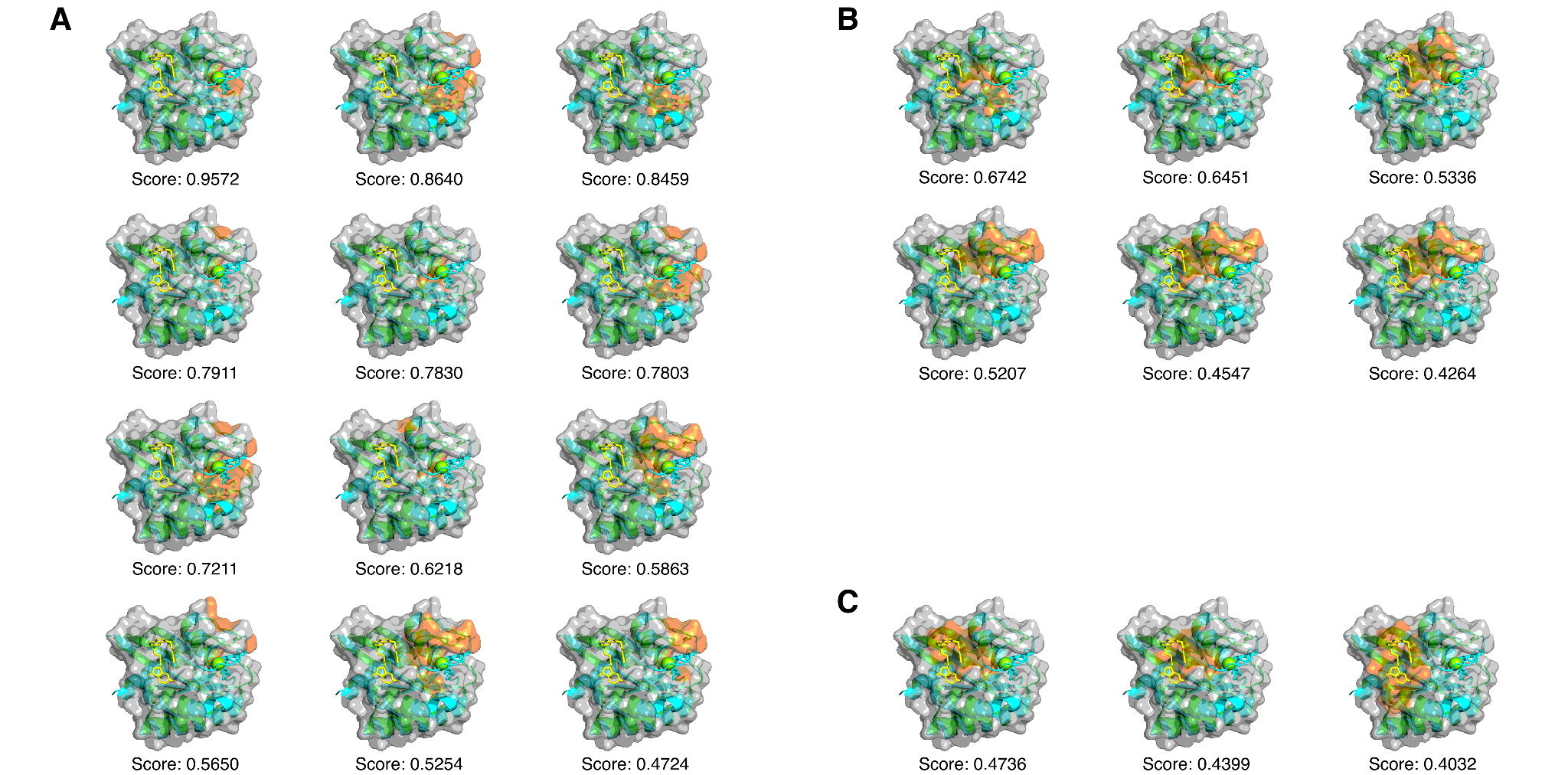


**Figure S12.** Individual (non-clustered) pockets on a GDP-bound KRAS structure (PDB ID: 5uk9). All pockets are either **A)** in the vicinity of the GDP/GTP binding site; **B)** between the GDP/GTP binding site and the switch I/II cryptic site; or **C)** in the vicinity of the switch I/II cryptic site. The *hotpocketNN* score (using Feature Set B, ESM2 embeddings only) for each individual pocket is displayed.
